# Sea Cucumber Peptides Ameliorate DSS-Induced Ulcerative Colitis: The Role of the Gut Microbiota, the Intestinal Barrier, and Macrophage Polarization

**DOI:** 10.3390/nu15224813

**Published:** 2023-11-17

**Authors:** Song Yu, Haixiang Guo, Zhonghao Ji, Yi Zheng, Bingbing Wang, Qingqing Chen, Hongyu Tang, Bao Yuan

**Affiliations:** 1 Department of Laboratory Animals, College of Animal Sciences, Jilin University, Changchun 130062, China; yusong22@mails.jlu.edu.cn (S.Y.); hxguo23@mails.jlu.edu.cn (H.G.); jizh21@mails.jlu.edu.cn (Z.J.); zhengyi@mail.jlu.edu.cn (Y.Z.); wangbb23@mails.jlu.edu.cn (B.W.); chenqq9919@mails.jlu.edu.cn (Q.C.); 2 Department of Basic Medicine, Changzhi Medical College, Changzhi 046000, China

**Keywords:** sea cucumber peptide, ulcerative colitis, gut microbiota, macrophage polarization

## Abstract

The incidence of ulcerative colitis (UC) is increasing annually. There are few treatments for UC patients, and some drugs have serious side effects. Sea cucumber peptide (SCP) has anti-inflammatory, antioxidant and other biological activities, and various sea cucumber species are in pharmaceutical development. However, relevant studies on the effects of SCP on UC progression are still lacking. In this study, a mouse model of acute colitis was induced by 3% dextran sulfate (DSS), and the effect of 500 mg/kg SCP on colitis was investigated. The results showed that SCP can alleviate DSS-induced colon damage and intestinal barrier damage. SCP significantly inhibited the expression of inflammatory factors and oxidative stress in UC mice. SCP reversed the intestinal microbiota dysregulation induced by DSS, inhibited the growth of *Sutterella*, *Prevotella_9* and *Escherichia-Shigella* harmful bacteria, and increased the abundance of *Lachnospiraceae_NK4A136_group*. At the same time, SCP treatment significantly inhibited the LPS-induced polarization of M1 macrophages, which may be mediated by two monopeptides, IPGAPGVP and TGPIGPPGSP, via FPR2. In conclusion, SCP can protect against colitis by modulating the intestinal microbiota composition and the intestinal barrier and inhibiting the polarization of M1 macrophages.

## 1. Introduction

Ulcerative colitis (UC) is a type of inflammatory bowel disease (IBD) that begins in the rectum and gradually spreads to the colon [1]. UC is a chronic disease characterized by high morbidity, severe symptoms, and a pronounced risk of relapse [2]. Most of the pathogenetic features are characterized by damage to the intestinal mucosa and the development of an inflammatory response with bloody stools, diarrhea, and weight loss [3]. The pathogenesis of UC is caused by a variety of factors, including genetic, environmental, and immunologic factors [4], but its pathogenesis is still unclear. The incidence and prevalence of UC has increased globally over time [5]. Currently, 15% of clinical patients require proctocolectomy, whereas patients with milder symptoms are treated with pharmacologic therapy consisting primarily of 5-aminosalicylic acid (5-ASA), glucocorticoids, or related immunomodulators [6]. However, prolonged use of these drugs can cause side effects and develop drug resistance [7]. Therefore, the development of a long-acting, affordable drug with a few side effects is of great importance for the treatment of clinical colitis.

The gastrointestinal barrier is important for maintaining the stability of the gastrointestinal tract, and when the intestinal epithelium is massively disrupted or dysregulated, it leads to a series of pathological processes, including damage to the epithelial barrier, the generation of inflammation, and microbial alterations in the gut [8]. Related studies have shown that disruption of the intestinal barrier is usually accompanied by an inflammatory response in both patients with UC and mice with DSS-induced colitis [9]. Disruption of the intestinal barrier leads to the entry of bacteria or harmful metabolites in the bloodstream, which in turn stimulates the body’s immune system, leading to the release of several inflammatory factors, such as IL-1β, IL-6, and TNF-α, which promote the development of inflammation [10,11]. In the colon, macrophages act as key immune cells that modulate intestinal inflammation and maintain mucosal homeostasis [12,13]. Macrophages are important components of the body’s immune system and play an important role in intestinal immune homeostasis [14]. Macrophages play different roles in the immune process depending on the type of polarization, and M1 macrophages are usually induced by LPS and are characterized by, among other things, a high expression of inducible nitric oxide synthase (iNOS) and the secretion of proinflammatory cytokines [15]. Restoring immune homeostasis by targeting macrophage polarization is an important therapeutic approach for UC [16,17,18]. In addition, disruption of the gut barrier usually leads to dysbiosis of the gut microbiota [19]. Relevant studies have shown that a balanced gut microbiota maintains intestinal barrier integrity and stimulates the body’s normal immune system [20]. Modulating the structure of the gut microbiota is likewise an effective treatment for alleviating UC [21]. The dextran sodium sulfate (DSS)-induced UC mouse model is similar to that of UC patients and is one of the most commonly used models in UC research [22,23,24]. Therefore, in the present study, we constructed an animal model of UC mice using DSS to investigate the modulatory effects of SCP on ulcerative colitis in terms of intestinal barrier, macrophage metabolism, and intestinal microbiota.

There is growing evidence that peptides may have a potential function in the treatment of clinical colitis [25,26]. For example, atrial natriuretic peptide (ANP) and its receptor, which is lowly expressed in the serum of UC patients and UC mice, can alleviate colitis by repairing intestinal barrier damage [27]. Human β-defensin 2 (hBD-2), an antimicrobial peptide, is largely under-expressed in the normal colon, but its expression is significantly increased in patients with UC [28], suggesting that hBD-2 may play an important role in UC progression. Sea cucumber peptide (SCP) is bioactive and prepared by microbial fermentation, chemical hydrolysis and enzymatic hydrolysis [29]. The composition of SCP includes many types of nonessential and essential amino acids, which means that SCP may have a wide range of potential biological activities [29]. SCP has been shown to inhibit the mRNA expression of inflammatory factors in Raw 264.7 cells due to LPS induction; thus, SCP has significant anti-inflammatory activity [3]. In addition, SCP possesses various biological properties [30], such as antioxidant, immunomodulatory, and anti-fatigue properties [31]. Currently, due to the mild therapeutic effects of dietary bioactive peptides, increasing efforts have been devoted to harnessing the pharmacological functions of SCP, which have been validated by molecular and animal experiments for many diseases [32,33]. For example, sea cucumber-derived peptide (FYDWPK) was able to improve cognitive impairment by preventing hippocampal cholinergic dysfunction and neuronal cell death [34]. Sea cucumber peptide ameliorates estrous cycle disorders and hormonal dysregulation in premature ovarian failure (POF) mice through activation of the cAMP signaling pathway [35]. However, there is a paucity of studies on the role of SCP in the progression of colitis and its mechanism.

In this study, an animal model of acute colitis in mice was established using 3% DSS, and the effect of SCP (500 mg/kg) on the progression of colitis in mice was investigated by determining the changes in body weight, DAI scores, the degree of colonic damage, the levels of inflammatory factors, and oxidative stress in each group of mice. The composition of the gut microbiota in different groups of mice was analyzed using 16S rRNA sequencing to investigate the role of the gut microbiota in the alleviation of colitis in mice by SCP. In vitro, LPS was used to induce inflammatory responses in Raw 264.7 cells to explore the specific mechanism by which SCP alleviates colitis in mice. In conclusion, the results of this study provide a theoretical basis for the use of SCP in drug development and disease prevention.

## 2. Materials and Methods

### 2.1. Animal Experiments

Six-week-old male BALB/c mice were purchased from Liaoning Changsheng Biotechnology Company Limited (Shenyang, China). This study was approved by the Laboratory Animal Center of Jilin University (license number: SY202307001).

Mice were randomly divided into the following 3 groups (N = 8) and acclimatized in an SPF environment for 7 days: the NC control group, the DSS model group and the SCP treatment group. The NC group was administered 0.9% NaCl via oral gavage for 21 days with free access to water. The DSS group was given 3% DSS (160110, MP Biomedicals, Irvine, CA, USA) in drinking water for 21 days to induce colitis. The SCP group was administered 500 mg/kg SCP (Wuhan Tiantianhao Bioproducts Co., Ltd., Wuhan, China) via oral gavage for 21 days; during the first 14 days, they were given water ad libitum, and during the second 7 days, they were given water containing 3% DSS ad libitum. Body weight was recorded daily during the experiment. The mice were sacrificed on day 21, the colon was clipped, and the length of the colon was measured, along with the DAI score based on body weight, colon length, and the degree of blood in the stool.

### 2.2. Clinical Scores and Histologic Analysis

The disease activity index (DAI) score is assessed based on body weight, stool characteristics, and bleeding, according to an established scoring system [10]. A portion of the colon was isolated for hematoxylin-eosin staining (H&E) and Alcian blue (AB) and periodic acid Schiff (PAS) sections. First, colon tissues were fixed in 10% paraformaldehyde solution and then routinely paraffin-embedded and sliced into thin sections of approximately 4 µm for H&E and AB-PAS staining; the sections were used to observe the internal histology of the intestinal tract and changes in cupped cells within the intestinal barrier.

### 2.3. RNA Extraction and RT–qPCR Analysis

A Total RNA Extraction Kit (SM130, Sevenbio, Beijing, China) was used to extract total RNA from the colon. RNA concentration and quality were measured using a NanoDrop2000 spectrophotometer. RNA was reverse transcribed into cDNA using cDNA Reverse Transcription Reagent, according to the manufacturer’s instructions (Monad, Suzhou, China). cDNA was quantified using the Fluorescence Quantification Reagent (Monad, Suzhou, China), according to the manufacturer’s instructions. All values were normalized to endogenous GAPDH. The expression of the relevant inflammatory factors IL-6, IL-1β, and TNF-α was calculated based on the comparative threshold cycling method. All sequences of primers used for RT–qPCR can be found in Supplementary Table S1.

### 2.4. Western Blotting

Proteins were extracted by adding colon tissues into protein lysate (RIPA) containing protease inhibitors (Yamei, Shanghai, China) and homogenized; the samples were then centrifuged to obtain the supernatant. The protein concentration was determined using a BCA protein kit (Biyun Tian, Shanghai, China). An appropriate amount of protein buffer was added and the samples were incubated at 95 °C for 10 min. A 10% PAGE gel (Yamei, Shanghai, China) was prepared according to the manufacturer’s instructions, and an appropriate amount of the sample was applied for gel electrophoresis to separate the proteins. Then, the desired proteins were transferred to PVDF membranes and the membranes were blocked with a rapid blocking solution and treated overnight at 4 °C with a primary antibody. Then, after washing with TBST, incubation was performed with secondary antibodies. Finally, the protein bands were visualized by using the Ultra-Sensitive Chemiluminescence Detection Kit (Yamei, Shanghai, China) and analyzed using ImageJ (V1.8.0) software. Endogenous GAPDH was utilized for band normalization. GAPDH antibody was purchased from Cell Signaling Technology (2118, Shanghai, China); Claudin-1, Occludin and ZO-1 primary antibodies were purchased from Affinity (AF0127, DF7504, AF5145, Changzhou, China).

### 2.5. Enzyme-Linked Immunosorbent Assays (ELISAs)

We homogenized the colon tissue and collected serum from mice. LPS, IL-6, IL-1β, TNF-α, MDA, SOD, and T-AOC levels were determined in colon tissues and serum according to the manufacturer’s instructions (SINOBESTBIO, Shanghai, China).

### 2.6. 16S rDNA Sequencing for Analysis of Gut Microbial Composition

After the mice were sacrificed, feces from the mouse colon were collected in sterile EP tubes, which were placed in liquid nitrogen and stored in a −80 °C freezer. Furthermore, the extraction of bacterial DNA from feces was performed according to the manufacturer’s instructions. The bacteria in the feces were sequenced using 16S rRNA sequencing based on the Illumina HiSeq platform (Lianchuan Bio, Hangzhou, China). Next, the data were filtered, and the nonrepetitive sequences were analyzed by clustering based on optional taxonomic units (OTUs) with 97% similarity. Based on the OTU data, the α-diversity and β-diversity of bacterial communities in each sample were calculated. A weighted UniFrac distance algorithm was combined with principal coordinate analysis (PCoA) and hierarchical cluster analysis to compare the structural differences of the bacterial communities in each sample.

### 2.7. Cell Culture and Treatment

The human colon cancer line HT-29 and Raw 264.7 cell were obtained from Boster Biologics (Wuhan, China). We used a cell-specific medium (Boster, Wuhan, China) for culturing that was maintained at 37 °C and 5% CO_2_.

The concentration of LPS (sigma)-treated cells was 100 ng/mL and the treatment time was 6 h.

### 2.8. Cell Viability Assay

Cells were inoculated in 96-well plates and treated with 0, 1, 2, 4, 8, or 16 mg/mL SCP for 24 h. The absorbance of individual wells at 450 nm was determined using the CCK8 kit (Meron, Dalian, China).

### 2.9. Flow Cytometric Analysis

Samples were collected 24 h after SCP treatment of cells, and LPS was added to treated cells 6 h before collection. Macrophages were collected by washing with PBS and centrifugation at 1000 rpm for 5 min; macrophages were then resuspended using flow-through a sampling buffer and subsequently stained and assayed on the instrument according to the manufacturer’s instructions [36]. After resuspension, APC anti-mouse F4/80 antibody and FITC anti-mouse CD11c antibody were added, and the samples were incubated for 30 min at 4 °C away from light, washed three times, and then subjected to flow cytometry (CytoFLEX, BECKMAN COULTER, Miami, FL, USA).

### 2.10. Analysis of Amino Acid Composition

The SCP concentration was determined by the BCA method. Hydrochloric acid was added to the sample to fully hydrolyze it, the sample was evaporated to dryness, and then 0.02 mol/L hydrochloric acid was added to resolubilize the sample, followed by filtering. Then, the sample was injected into the derivatization tube, and the boric acid buffer and derivatizing agent were added sequentially; the samples were heated in an oven at 55 °C for 10 min before liquid–phase injection.

### 2.11. SCP Identification

The samples were desalted using ZipTip C18. Next, the peptides were solubilized with a lysis solution containing 0.1% formic acid and 5% acetonitrile, and finally, after centrifugation at 13,500 rpm and 4 °C for 20 min, the supernatant was aspirated for SCP identification.

The parameters of liquid chromatography were set as follows: mobile phase A: 0.1% formic acid; mobile phase B: 0.1% formic acid, 80% CAN; the samples were separated by the following gradient: 3–8% B over 4 min, 8–32% B over 39 min, 32–44% B over 5 min, and 44–99% B over 5 min. The resolution of the primary mass spectrometry (PMS) was 120,000, and the scanning range was 350–1550 mz. The resolution of the secondary mass spectra was 30,000, and the results were compared with the data in PEAKS Studio 11 software to analyze the mass spectrometry results.

### 2.12. Molecular Docking

The hydrogenation of proteins, the hydrogenation of small molecules and the determination of torsional bonds was performed using AutoDock Tools 1.5.6 and the result was saved as a pdbqt file. The molecular docking range parameters were set as follows: center_x = −28.953, center_y = 22.643, center_z = 2.398, size_x = 60.0, size_y = 62.0, and size_z = 72.0. The docking method was set to semiflexible docking and the docking algorithm was set to Lamarckian Genetic Algorithm using the Grid board. AutoDock Vina2.3.0 was run to determine the docking binding free energy and generate the docking result file.

### 2.13. Statistical Analysis

All data were statistically analyzed using GraphPad Prism 9.8 software, and the data are expressed as the mean ± standard error (SEM). Statistical differences were analyzed using one-way ANOVA. * *p* < 0.05 was considered statistically significant.

## 3. Results

### 3.1. SCP Ameliorates DSS-Induced Colitis Symptoms in Mice

**Figure 1:**
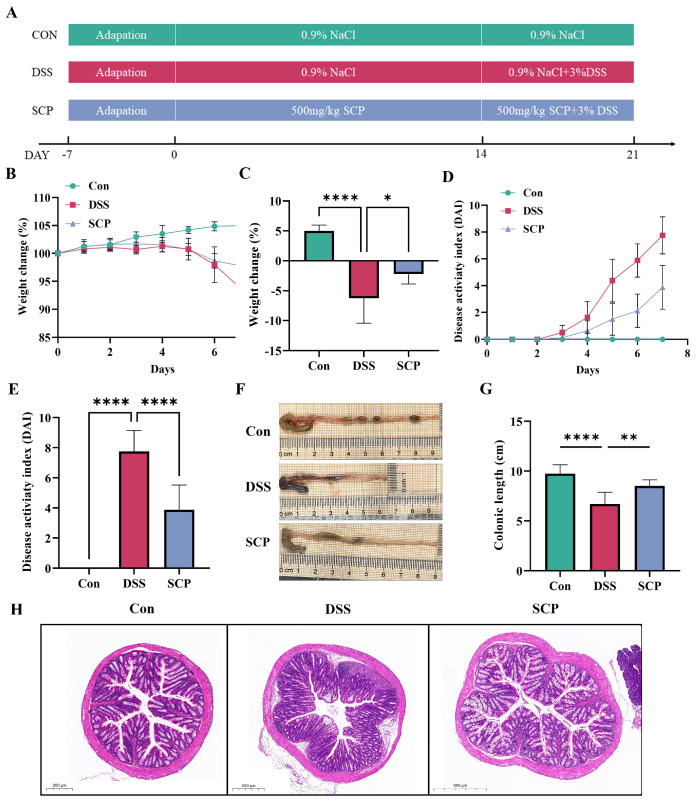
Effect of SCP on colitis model mice. (**A**) Establishment of a mouse model of enteritis. (**B**) Body weight changes in mice after 7 days of modeling. (**C**) Comparison of changes in body weight on day 7 between different groups. (**D**) DAI scores of mice at 7 days of modeling. (**E**) Comparison of changes in DAI scores on day 7 between different groups. (**F**) Schematic diagram of the length of the colon. (**G**) Comparison of colon length between different groups. (**H**) H&E staining between different groups. Data are shown as the mean ± SEM (*n* = 8), * *p* < 0.05, ** *p* < 0.01, **** *p* < 0.0001.

To assess the role of SCP in the progression of colitis in UC mice, a mouse model of acute colitis was established in this study using 3% DSS (Figure 1A). In the last seven days of DSS modeling, the body weight of the DSS group decreased significantly compared with that of the control group, and SCP alleviated the trend of body weight loss seen in the DSS group (Figure 1B). On the last day of modeling, SCP significantly mitigated the decline seen in the DSS group (Figure 1C). DAI scores in the DSS group increased significantly over time, whereas DAI scores in the SCP group decreased (Figure 1D), and the differences were significant on the last day (Figure 1E). The colon length was significantly shorter after DSS treatment; however, SCP treatment restored the colon length in mice (Figure 1F,G). Meanwhile, histologic results showed that in the nodes of DSS-induced colitis mice, epithelial crypts disappeared, cuprocytes were reduced, the mucosal barrier was impaired, and the infiltration of inflammatory cells was observed; in contrast, the pathological changes in the nodes were ameliorated by SCP (Figure 1H). These results suggest that SCP can ameliorate DSS-induced colitis.

### 3.2. SCP Alleviates Colonic Inflammation in UC Mice

**Figure 2:**
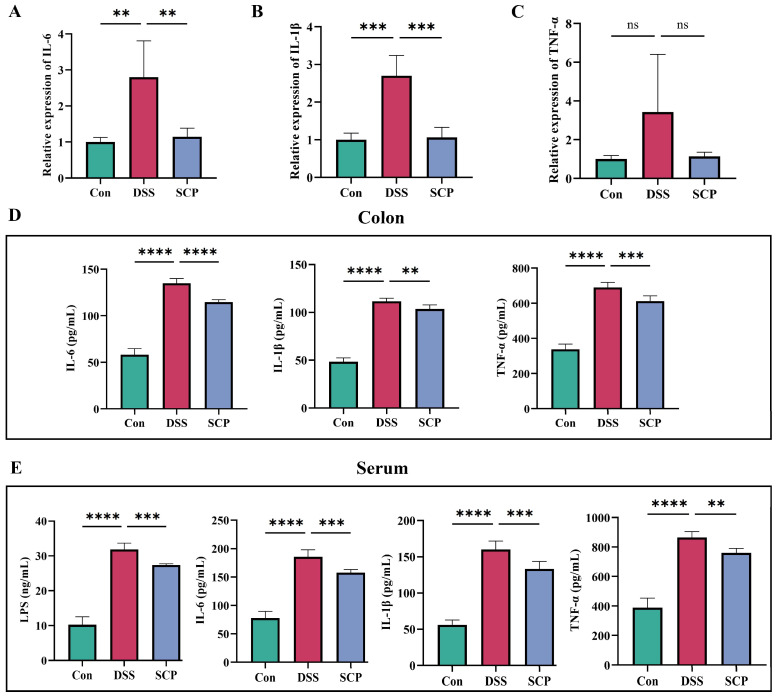
Effect of SCP on colonic inflammation in mice. (**A**–**C**) Expression of IL-6, IL-1β, and TNF-α mRNA in colonic tissues. (**D**) Secretion of IL-6, IL-1β and TNF-α in colon homogenates. (**E**) Secretion of IL-6, IL-1β and TNF-α in mouse serum. Data are shown as the mean ± SEM (*n* = 3), ns, *p* > 0.5, ** *p* < 0.01, *** *p* < 0.001, **** *p* < 0.0001.

Injury to the intestines of mice with DSS-induced colitis is closely related to inflammation, so in the present study, we examined the expression of numerous cellular inflammatory factors in the colon and serum of mice. The results showed that the DSS group had significantly elevated mRNA expression of the inflammatory factors IL-6 and IL-1β, while SCP inhibited the synthesis of inflammatory factors (Figure 2A,B). Inflammatory factors in colon homogenates and the serum were also examined, and IL-6 and IL-1β levels were significantly elevated in colon tissues in the DSS model group. SCP treatment also significantly reduced the levels of IL-6, IL-1β, and TNF-α (Figure 2D). In serum, IL-6, IL-1β, and TNF-α levels were significantly increased in the DSS group of mice compared with that in the control group. SCP treatment significantly reduced the levels of IL-6, IL-1β, and TNF-α-related inflammatory factors (Figure 2E). Although the difference in the mRNA expression of TNF-α was not significant (Figure 2C), it had the same trend as that of the other two inflammatory factors. The above results suggest that SCP can reduce the levels of inflammatory factors in the colons of UC mice.

### 3.3. SCP Alleviates DSS-Induced Oxidative Stress

**Figure 3:**
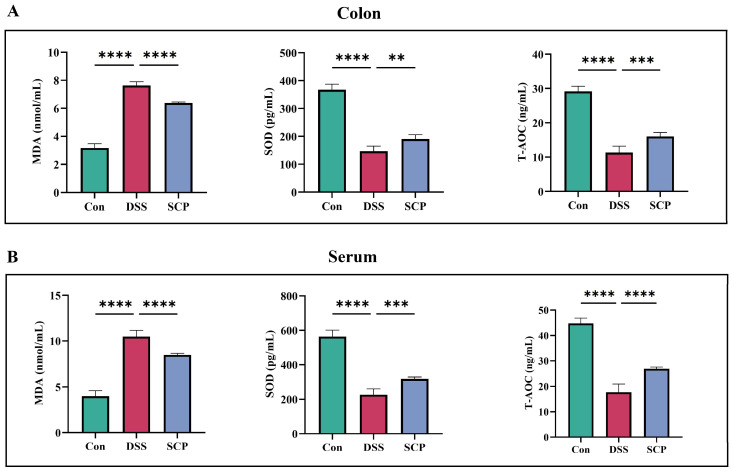
Effect of SCP on oxidative stress indicators in DSS-induced colitis mice. (**A**) Changes in indicators of oxidative stress in colon homogenates. (**B**) Changes in indices of oxidative stress in serum. Data are shown as the mean ± SEM (n = 3), ** *p* < 0.01, *** *p* < 0.001, **** *p* < 0.0001.

In the colon, DSS-induced enteritis was followed by an increase in MDA levels and a decrease in SOD and T-AOC levels, whereas the addition of SCP resulted in a significant decrease in MDA levels and a significant increase in SOD and T-AOC levels (Figure 3A). The results in serum and colon homogenates were consistent (Figure 3B). This shows that SCP can alleviate DSS-induced oxidative stress.

### 3.4. SCP Alleviates DSS-Induced Intestinal Barrier Damage

**Figure 4:**
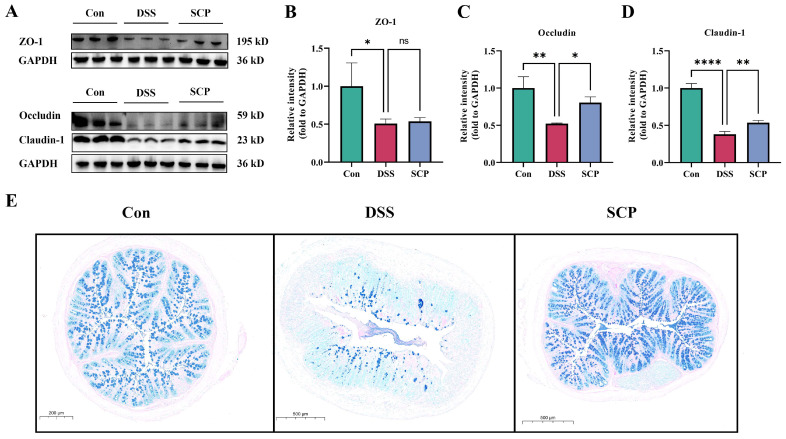
Effect of SCP on DSS-induced intestinal damage. (**A**) Representative Western blotting images of the tight junction proteins ZO-1, Occludin and Claudin-1. (**B**) Relative expression of the ZO-1 protein. (**C**) Relative expression of the occludin protein. (**D**) Relative expression of the claudin-1 protein. (**E**) AB-PAS staining of the colon samples from different groups. Data are shown as the mean ± SEM, ns *p* > 0.5, * *p* < 0.05, ** *p* < 0.01, **** *p* < 0.0001.

In this study, we evaluated cup cells in the colon by using AB-PAS staining. In colon tissue, cup cells were stained dark blue, while other areas were light blue or had no color. In the AB-PAS-stained sections, there was a significant reduction in cup cells and mucins in the colonic tissues of mice with DSS-induced colitis. We determined changes in tight junction proteins in the intestine by WB. The results showed that the ZO-1, Occludin and Claudin-1 proteins were decreased in the intestines of DSS-induced colitis mice, and both Occludin and Claudin-1 levels were significantly restored after SCP treatment (Figure 4A–D). Although the change in the ZO-1 protein was not significant, there was a similar trend (Figure 4A,B). The tissue sections showed that SCP alleviated the DSS-induced decrease in the number of cup cells and inhibited the mucin degradation, similar to what was seen in the control group (Figure 4E). Thus, SCP alleviated DSS-induced intestinal barrier damage by restoring the expression of tight junction proteins.

### 3.5. SCP Improves the Intestinal Flora Structure of Mice with DSS-Induced Colitis

**Figure 5:**
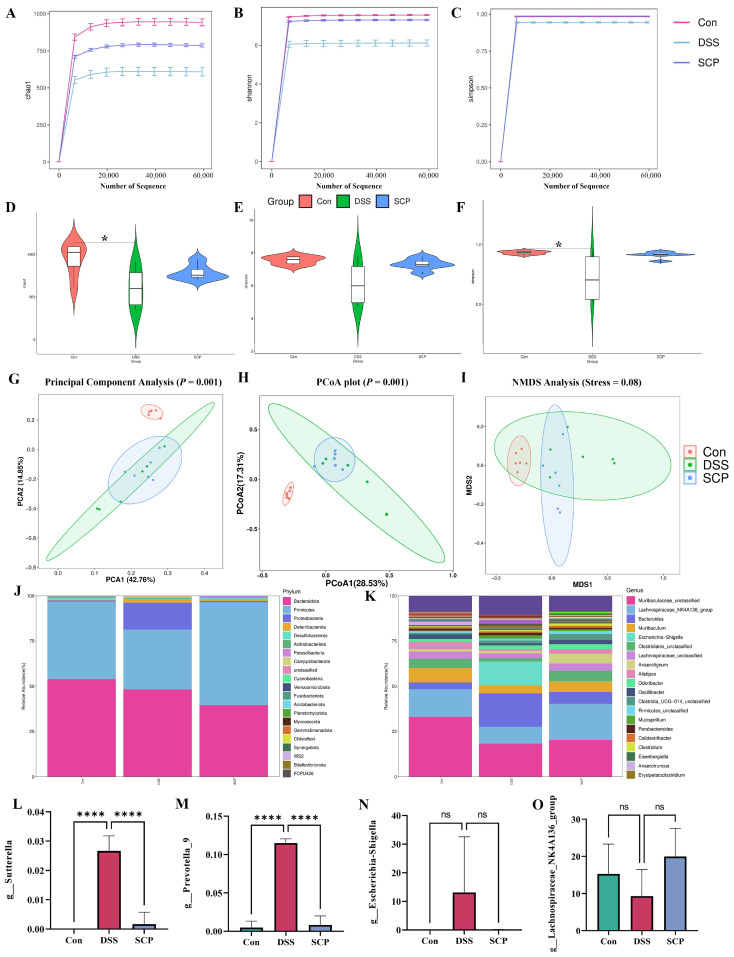
Effect of SCP on the intestinal flora of colitis mice. (**A**–**C**) Dilution curves the for Chao1, Shannon, and Simpson indices. (**D**–**F**) The Chao1, Shannon, and Simpson indices for alpha diversity analysis. (**G**–**I**) Beta diversity analysis. (**J**) Histogram of community distribution at the portal level. (**K**) Histogram of genus level community distribution. (**L**–**O**) The abundance of bacterial genera. Data are shown as the mean ± SEM (*n* = 6), ns *p* > 0.5, * *p* < 0.05, **** *p* < 0.0001.

The intestinal flora is a key factor that impacts the intestinal tract, so we investigated whether SCP alleviated DSS-induced colitis by altering the intestinal flora. We detected the microorganisms that were carried in mouse feces by 16S rRNA. The results showed that the amount of data for all samples met the sequencing requirements, the sequencing results were plausible, and the sparse curve stagnated with sequencing, indicating that diversity was well represented in all samples in this study. The abundance of intestinal flora in DSS-treated mice was significantly reduced, while SCP treatment significantly increased the abundance of intestinal flora in DSS-induced mice (Figure 5A–C). Meanwhile, in the DSS group, there was significantly reduced α-diversity compared to that of the control group, and although the Chao1 (Figure 5D) index showed that SCP had no effect on the α-diversity, Shannon’s index (Figure 5E) and Simpson’s index (Figure 5F) showed an improvement in the α-diversity of DSS mice by SCP.

Meanwhile, PCA, CoA and NMDS analyses showed that DSS treatment significantly altered the distribution of gut microbial communities, while SCP restored the structure of the gut microbiota (Figure 5G–I). In addition, we examined the distribution of gut microorganisms at the structural level and of bacteria at the phylum and genus levels (Figure 5J,K), both of which reverted to that of the control following SCP treatment. At the genus level, the abundance of *Sutterella*, *Prevotella_9*, and *Escherichia-Shigella* flora was increased, but the abundance of *Lachnospiraceae_NK4A136_group* flora was decreased compared with that of the control group; meanwhile, SCP restored the abundance of these four floras to that of the control mice (Figure 5L–O). In summary, SCP ameliorated the colonic gut microbiota disruption in UC mice.

### 3.6. Correlation Analysis Results

**Figure 6:**
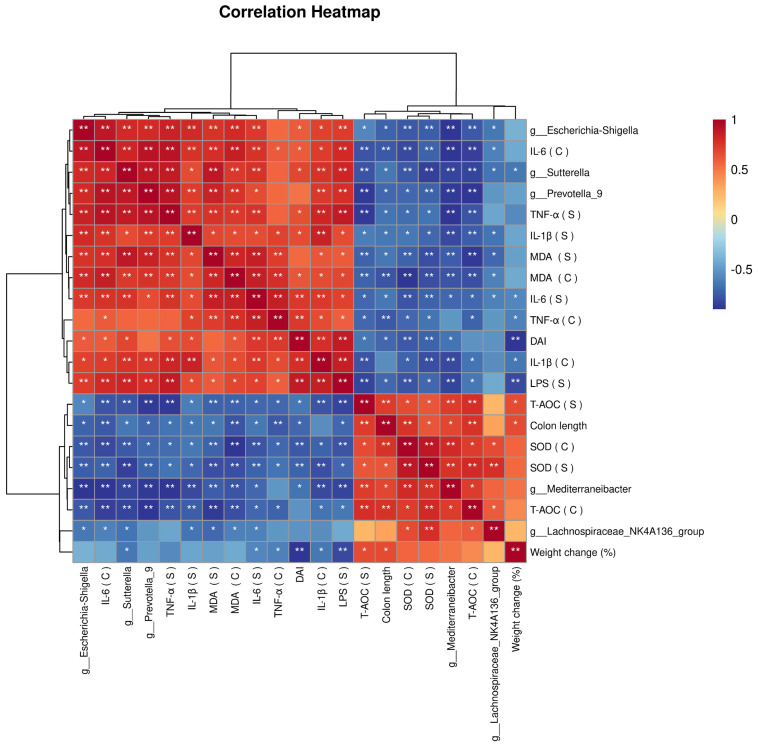
Correlation of the gut flora and UC indicators. Blue: negative correlation, red: positive correlation. * *p* < 0.05, ** *p* < 0.0001. Correlation network analysis was performed using the OmicStudio tools at https://www.omicstudio.cn/tool, accessed on 20 October 2023.

In this study, a correlation analysis of the flora and inflammatory factors was performed (Figure 6). The results showed that three harmful bacteria, *Escherichia-Shigella*, *Sutterella*, and *Prevotella_9*, were positively correlated with the inflammatory response, whereas two beneficial bacteria, *Mediterraneibacter* and *Lachnospiraceae_NK4A136_group*, were negatively correlated with the inflammatory response.

### 3.7. SCP Ameliorates Colonic Inflammation in Mice by Promoting Cell Proliferation and Modulating Macrophage Polarization

**Figure 7:**
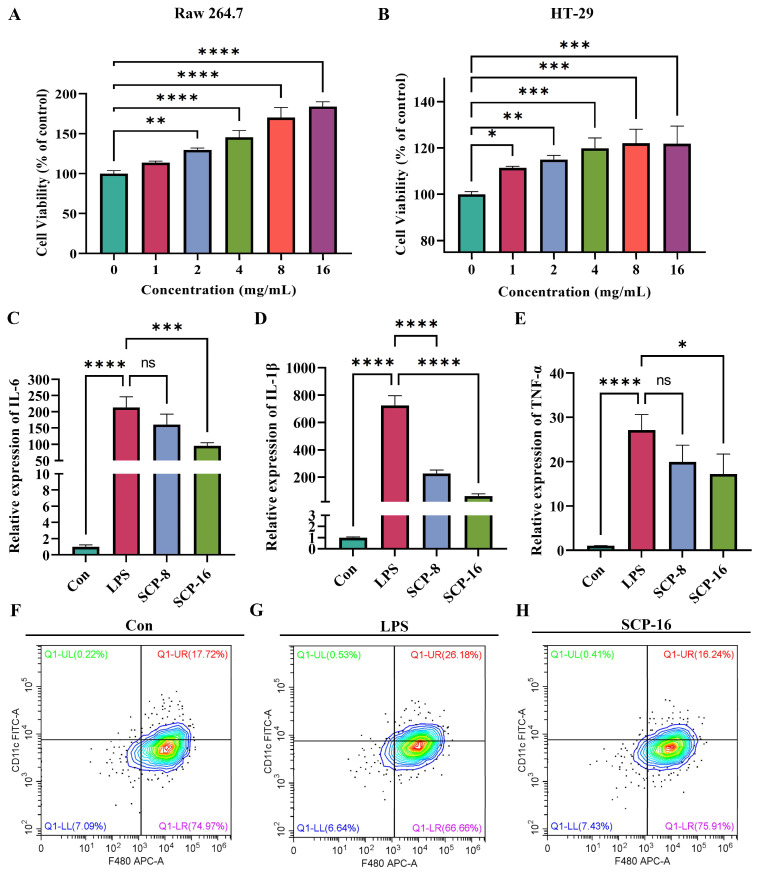
Effect of SCP on cell proliferation and macrophage polarization. (**A**,**B**) Detection of the viability of Raw 264.7 (left) and HT29 (right) cells. (**C**–**E**) Expression of the relevant inflammatory factors IL-6, IL-1β and TNF-α. (**F**–**H**) Graphs of macrophage polarization results in different treatment groups. All data are shown as the mean ± SEM, ns *p* > 0.5, * *p* < 0.05, ** *p* < 0.01, *** *p* < 0.001, **** *p* < 0.0001.

We examined the effect of different concentrations of SCP on the proliferation of Raw 264.7 and HT29 cells and found that SCP promoted the proliferation of both cell lines (Figure 7A,B). Following the treatment of cells with different concentrations of SCP, LPS successfully promoted cellular inflammation, while 16 mg/mL was able to significantly inhibit the expression of the relevant inflammatory factors IL-6, IL-1β and TNF-α (Figure 7C–E). We selected a concentration of 16 mg/mL for cell flow, and the results showed that LPS promoted macrophage polarization compared to that of the NC group, while 16 mg/mL SCP inhibited macrophage polarization (Figure 7F–H).

### 3.8. SCP Identification and the Molecular Docking of Single Peptides

**Figure 8:**
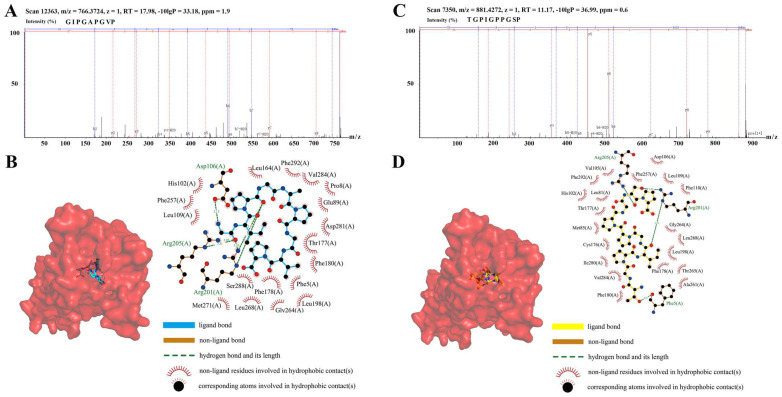
Identification of small peptides in SCP and molecular docking. (**A**) Mass spectra of the GIPGAPGVP small peptide. (**B**) Predicted molecular docking of GIPGAPGVP with FPR2. (**C**) Mass spectra of the TGPIGPPGSP small peptide. (**D**) Predicted molecular docking of TGPIGPPGSP with FPR2.

In this study, the amino acids in SCP were determined; SCP contained a total of 16 amino acids, and the most abundant amino acid was glycine. The detailed results are shown in Supplementary Table S2. We identified the small peptides of SCP species, and a total of 121 small peptides were detected. Finally, we chose the two small peptides with the highest ratios, i.e., GIPGAPGVP (Figure 8A) and TGPIGPPGSP (Figure 8C), for molecular docking with FPR2. The detailed results are shown in Supplementary Table S3.

In the GIPGAPGVP group, the peptide formed four hydrogen-bonds with the amino acid residues Asp106, Arg201, and Arg205 of the A-chain of the FPR2 protein and multiple hydrophobic bonds with surrounding amino acid residues (Figure 8B). In the TGPIGPPGSP group, the peptide formed four hydrogen-bonds with the amino acid residues Phe5, Arg201, and Arg205 of the A-chain of the FPR2 protein and multiple hydrophobic bonds with surrounding amino acid residues (Figure 8D). Four hydrogen-bonds and multiple hydrophobic bonds were formed between the peptide and the FPR2 protein in both sets of docking results, indicating that the peptide and the protein bound with hydrophilic–hydrophobic synergistic interactions; furthermore, the binding energies were all lower than −9 kcal/mol, which is a strong binding effect, so it is very likely that the addition of GIPGAPGVP and TGPIGPPGSP will affect the structure–function and biological activity of the FPR2 protein.

## 4. Discussion

Sea cucumber, as a marine organism, has long been used as a traditional tonic in many countries, and due to its rich functionality, a variety of sea cucumber species are being exploited in food and pharmaceutical development [29]. Recently, SCP has been shown to alleviate DSS-induced chronic colitis in mice by alleviating gut microbial dysbiosis and modulating the miR-155/SOCS1 axis [37]. However, the effects of SCP on UC inflammation and macrophage M1 polarization remain unclear. To investigate the mechanism related to SCP alleviation of colitis in UC mice, a UC mouse model was established by 3% DSS and mice were treated with SCP. Furthermore, the anti-inflammatory effects of SCP were tested and validated both in vivo and in vitro. Interestingly, SCP promoted the proliferation of Raw 264.7 and HT29 cells, and MSCs have been shown to alleviate the symptoms of DSS-induced colitis by promoting the regeneration of the colonic epithelium [38]. Thus, SCP ameliorated UC inflammatory responses by alleviating gut microbiota dysbiosis, maintaining intestinal barrier integrity and inhibiting macrophage M1 polarization. These findings suggest that SCP, as a mildly active dietary peptide, holds promise for application as a therapeutic agent in the treatment of UC.

UC is a prevalent chronic inflammatory disease whose symptoms include weight loss, diarrhea, and rectal bleeding [39]. In our results, mice in the DSS group showed weight loss, diarrhea, and rectal bleeding, whereas SCP treatment significantly alleviated DSS-induced symptoms in UC mice. Disruption of the intestinal barrier usually leads to the invasion of pathogenic microorganisms or toxins into the intestines, which is a key factor in the development of ulcerative colitis [40]. Related studies have shown that the intestinal barrier is mainly regulated by epithelial tight junction (TJ) proteins [41]. ZO-1, Occludin and Claudin proteins are important for maintaining the normal function of the intestinal barrier [42,43]. Disruption of the intestinal barrier is usually accompanied by the accumulation of inflammatory factors and reactive oxygen species [44]. In our findings, SCP significantly inhibited the secretion of TNF-α, IL-1β, and IL-6, and the expression of these proteins is considered to be the main pro-inflammatory cytokines that exacerbate inflammatory responses [45]. In addition, SCP treatment upregulated the expression of colonic tight junction proteins (ZO-1, Occludin and Claudin-1) in UC mice. These results suggest that SCP can alleviate DSS-induced colitis by maintaining the structure and integrity of the intestinal barrier.

Dysbiosis of the gut microbiota is strongly associated with the development of UC, and gut microbiota diversity is reduced in patients with UC [46]. Furthermore, DSS-induced UC in mice results in an altered gut microbiota [47,48]. Decreases in the phylum Firmicutes and increases in Proteobacteria are the main features of UC ecological dysbiosis [46]. In our study, SCP treatment significantly increased the abundance of the phylum Firmicutes and significantly decreased the abundance of Proteobacteria. The abundances of three harmful bacteria, *Sutterella*, *Prevotella_9* and *Escherichia-Shigella*, were significantly increased by DSS treatment. The above three genera have been shown to be significantly increased following DSS treatment [49,50,51]. Meanwhile, SCP reduced the expression of these harmful bacteria. *Lachnospiraceae_NK4A136_group* is a beneficial bacterium, and several studies have found that DSS inhibits its growth and therapeutic drugs promote its growth [51,52]; SCP treatment similarly promotes the expression of beneficial bacteria. These results indicated that SCP restored the balance of intestinal microorganisms by regulating the intestinal microbiota and significantly alleviated DSS-induced colitis in mice.

During the progression of UC, disruption of the gut microbiota affects the body’s immune system [53]. Macrophages play an important role in maintaining intestinal immune homeostasis [54]. Macrophage polarization is an important strategy for the treatment of UC [55], and in vitro LPS is commonly used to treat resting macrophages, which leads to proinflammatory differentiation and promotes the secretion of inflammatory factors (TNF-α, IL-6, and IL-1β). In this study, the results of animal experiments showed that SCP significantly suppressed the expression of inflammatory factors and oxidative stress in DSS-induced mice. M1 polarization was induced in RAW 264.7 cells using LPS, and SCP was found to significantly inhibit macrophage M1 polarization and the expression of inflammatory factors. We also characterized the amino acid composition of SCP and showed that the three most abundant amino acids in SCP were proline, glycine, and glutamate. This is consistent with the results of a previous report [29]. At the same time, we characterized the peptide composition of SCP and selected two single peptides with a relatively high abundance and activity for molecular docking experiments; the results showed that both single peptides were able to bind with FPR2. Furthermore, FPR2 can regulate the inflammatory response through macrophage polarization [56,57]. Thus, SCP may regulate macrophage M1 polarization through FPR2 and thus exert its anti-inflammatory effects.

## 5. Conclusions

In this study, we showed that SCP treatment significantly reduced the weight change and DAI score of mice and significantly inhibited DSS-induced inflammation and oxidative stress. SCP alleviated DSS-induced colon damage by reducing intestinal microbiome dysregulation and repairing the intestinal barrier. In addition, SCP treatment suppressed inflammation levels in vitro by inhibiting macrophage M1 polarization, which may be achieved by FPR2. In subsequent studies, we will explore the specific mechanism by which SCP alleviates UC from the aspect of functional verification of the identified single peptide molecules. In summary, SCP, as a foodborne polypeptide with a variety of biological activities, has the potential to be used as an alternative in the clinical treatment of UC.

Interestingly, SCP treatment significantly promoted the proliferation of Raw 264.7 and HT29 cells in this study. MSCs have been shown to alleviate UC symptoms by promoting colonic epithelial integrity and regeneration [38]. The above evidence suggests that SCP may also alleviate the symptoms of DSS-induced colitis by promoting colonic epithelial regeneration, which is a hypothesis we need to test in the future.

## Data Availability

Data are contained within the article.

## Supplementary Materials

The following supporting information can be downloaded at: https://www.mdpi.com/article/10.3390/nu15224813/s1, Table S1: All primers; Table S2: Amino acid composition of SCP; Table S3: Peptide identification of SCP.

## References

[1] Park S.C., Jeen Y.T. (2015). Current and emerging biologics for ulcerative colitis. Gut Liver..

[2] Zhang L., Yao X., Ma M., Ding Y., Zhang H., He X., Song Z. (2021). Protective Effect of l-Theanine against DSS-Induced Colitis by Regulating the Lipid Metabolism and Reducing Inflammation via the NF-kappaB Signaling Pathway. J. Agric. Food Chem..

[3] Ahmed I., Roy B.C., Khan S.A., Septer S., Umar S. (2016). Microbiome, Metabolome and Inflammatory Bowel Disease. Microorganisms.

[4] Li M.X., Li M.Y., Lei J.X., Wu Y.Z., Li Z.H., Chen L.M., Zhou C.L., Su J.Y., Huang G.X., Huang X.Q. (2022). Huangqin decoction ameliorates DSS-induced ulcerative colitis: Role of gut microbiota and amino acid metabolism, mTOR pathway and intestinal epithelial barrier. Phytomedicine.

[5] Yang M., Zhang Q., Taha R., Abdelmotalab M.I., Wen Q., Yuan Y., Zhao Y., Li Q., Liao C., Huang X. (2022). Polysaccharide from Atractylodes macrocephala Koidz. ameliorates DSS-induced colitis in mice by regulating the Th17/Treg cell balance. Front. Immunol..

[6] Cosnes J., Gower-Rousseau C., Seksik P., Cortot A. (2011). Epidemiology and natural history of inflammatory bowel diseases. Gastroenterology.

[7] Singh D., Srivastava S., Pradhan M., Kanwar J.R., Singh M.R. (2015). Inflammatory Bowel Disease: Pathogenesis, Causative Factors, Issues, Drug Treatment Strategies, and Delivery Approaches. Crit. Rev. Ther. Drug Carrier Syst..

[8] Patankar J.V., Becker C. (2020). Cell death in the gut epithelium and implications for chronic inflammation. Nat. Rev. Gastroenterol. Hepatol..

[9] Zhong Y., Liu W., Xiong Y., Li Y., Wan Q., Zhou W., Zhao H., Xiao Q., Liu D. (2022). Astragaloside Ⅳ alleviates ulcerative colitis by regulating the balance of Th17/Treg cells. Phytomedicine.

[10] Yadav P.N., Liu Z., Rafi M.M. (2003). A diarylheptanoid from lesser galangal (*Alpinia officinarum*) inhibits proinflammatory mediators via inhibition of mitogen-activated protein kinase, p44/42, and transcription factor nuclear factor-kappa B. J. Pharmacol. Exp. Ther..

[11] Gallimore A.M., Godkin A. (2013). Epithelial barriers, microbiota, and colorectal cancer. N. Engl. J. Med..

[12] Gren S.T., Grip O. (2016). Role of Monocytes and Intestinal Macrophages in Crohn’s Disease and Ulcerative Colitis. Inflamm. Bowel Dis..

[13] Yang Z., Lin S., Feng W., Liu Y., Song Z., Pan G., Zhang Y., Dai X., Ding X., Chen L. (2022). A potential therapeutic target in traditional Chinese medicine for ulcerative colitis: Macrophage polarization. Front. Pharmacol..

[14] Xie Y., Yu L., Cheng Z., Peng Y., Cao Z., Chen B., Duan Y., Wang Y. (2022). SHED-derived exosomes promote LPS-induced wound healing with less itching by stimulating macrophage autophagy. J. Nanobiotechnol..

[15] Shayan M., Padmanabhan J., Morris A.H., Cheung B., Smith R., Schroers J., Kyriakides T.R. (2018). Nanopatterned bulk metallic glass-based biomaterials modulate macrophage polarization. Acta Biomater..

[16] Wu M.M., Wang Q.M., Huang B.Y., Mai C.T., Wang C.L., Wang T.T., Zhang X.J. (2021). Dioscin ameliorates murine ulcerative colitis by regulating macrophage polarization. Pharmacol. Res..

[17] Hoffner O’Connor M., Berglind A., Kennedy Ng M.M., Keith B.P., Lynch Z.J., Schaner M.R., Steinbach E.C., Herzog J., Trad O.K., Jeck W.R. (2022). BET Protein Inhibition Regulates Macrophage Chromatin Accessibility and Microbiota-Dependent Colitis. Front. Immunol..

[18] De Matteis R., Flak M.B., Gonzalez-Nunez M., Austin-Williams S., Palmas F., Colas R.A., Dalli J. (2022). Aspirin activates resolution pathways to reprogram T cell and macrophage responses in colitis-associated colorectal cancer. Sci. Adv..

[19] Costello S.P., Hughes P.A., Waters O., Bryant R.V., Vincent A.D., Blatchford P., Katsikeros R., Makanyanga J., Campaniello M.A., Mavrangelos C. (2019). Effect of Fecal Microbiota Transplantation on 8-Week Remission in Patients with Ulcerative Colitis: A Randomized Clinical Trial. JAMA.

[20] Cheng J., Liu D., Huang Y., Chen L., Li Y., Yang Z., Fu S., Hu G. (2023). Phlorizin Mitigates Dextran Sulfate Sodium-Induced Colitis in Mice by Modulating Gut Microbiota and Inhibiting Ferroptosis. J. Agric. Food Chem..

[21] Han D., Wu Y., Lu D., Pang J., Hu J., Zhang X., Wang Z., Zhang G., Wang J. (2023). Polyphenol-rich diet mediates interplay between macrophage-neutrophil and gut microbiota to alleviate intestinal inflammation. Cell Death Dis..

[22] Zhang J., Chen L., Xu Q., Zou Y., Sun F., Zhou Q., Luo X., Li Y., Chen C., Zhang S. (2023). Ubc9 regulates the expression of MHC II in dendritic cells to enhance DSS-induced colitis by mediating RBPJ SUMOylation. Cell Death Dis..

[23] He P., Zhang Y., Chen R., Tong Z., Zhang M., Wu H. (2023). The maca protein ameliorates DSS-induced colitis in mice by modulating the gut microbiota and production of SCFAs. Food Funct..

[24] Zhang D., Ge F., Ji J., Li Y.J., Zhang F.R., Wang S.Y., Zhang S.J., Zhang D.M., Chen M. (2023). beta-sitosterol alleviates dextran sulfate sodium-induced experimental colitis via inhibition of NLRP3/Caspase-1/GSDMD-mediated pyroptosis. Front. Pharmacol..

[25] Huang Y., Zheng Y., Yang F., Feng Y., Xu K., Wu J., Qu S., Yu Z., Fan F., Huang L. (2022). *Lycium barbarum* Glycopeptide prevents the development and progression of acute colitis by regulating the composition and diversity of the gut microbiota in mice. Front. Cell Infect. Microbiol..

[26] Sun X., Huang Y., Zhang Y.L., Qiao D., Dai Y.C. (2020). Research advances of vasoactive intestinal peptide in the pathogenesis of ulcerative colitis by regulating interleukin-10 expression in regulatory B cells. World J. Gastroenterol..

[27] Chen C., Zhang Y., Tao M., Zhao X., Feng Q., Fei X., Fu Y. (2022). Atrial Natriuretic Peptide Attenuates Colitis via Inhibition of the cGAS-STING Pathway in Colonic Epithelial Cells. Int. J. Biol. Sci..

[28] Wehkamp J., Fellermann K., Herrlinger K.R., Baxmann S., Schmidt K., Schwind B., Duchrow M., Wohlschlager C., Feller A.C., Stange E.F. (2002). Human beta-defensin 2 but not beta-defensin 1 is expressed preferentially in colonic mucosa of inflammatory bowel disease. Eur. J. Gastroenterol. Hepatol..

[29] Lu Z., Sun N., Dong L., Gao Y., Lin S. (2022). Production of Bioactive Peptides from Sea Cucumber and Its Potential Health Benefits: A Comprehensive Review. J. Agric. Food Chem..

[30] Yue H., Tian Y., Li Y., Bai X., Wang X., Wang Y., Li Z., Xue C., Wang J. (2022). Comparative study of holothurin A and echinoside A on inhibiting the high bone turnover via downregulating PI3K/AKT/beta-catenin and OPG/RANKL/NF-kappaB signaling in ovariectomized mice. Food Funct..

[31] Gong P.X., Wang B.K., Wu Y.C., Li Q.Y., Qin B.W., Li H.J. (2020). Release of antidiabetic peptides from Stichopus japonicas by simulated gastrointestinal digestion. Food Chem..

[32] Mao J., Zhang Z., Chen Y., Wu T., Fersht V., Jin Y., Meng J., Zhang M. (2021). Sea cucumber peptides inhibit the malignancy of NSCLC by regulating miR-378a-5p targeted TUSC2. Food Funct..

[33] Li Y., Xu J., Su X. (2017). Analysis of Urine Composition in Type II Diabetic Mice after Intervention Therapy Using Holothurian Polypeptides. Front. Chem..

[34] Zhao Y., Lu Z., Xu X., Sun N., Lin S. (2022). Sea Cucumber-Derived Peptide Attenuates Scopolamine-Induced Cognitive Impairment by Preventing Hippocampal Cholinergic Dysfunction and Neuronal Cell Death. J. Agric. Food Chem..

[35] Luo X., Liu W., Zhao M., Wang J., Gao X., Feng F. (2023). The evaluation of sea cucumber (*Acaudina leucoprocta*) peptide on sex hormone regulation in normal and premature ovarian failure female mice. Food Funct..

[36] Jiao Y., Zhang T., Zhang C., Ji H., Tong X., Xia R., Wang W., Ma Z., Shi X. (2021). Exosomal miR-30d-5p of neutrophils induces M1 macrophage polarization and primes macrophage pyroptosis in sepsis-related acute lung injury. Crit. Care.

[37] Mao J., Zhao Y., Wang L., Wu T., Jin Y., Meng J., Zhang M. (2023). Sea Cucumber Peptide Alleviates Ulcerative Colitis Induced by Dextran Sulfate Sodium by Alleviating Gut Microbiota Imbalance and Regulating miR-155/SOCS1 Axis in Mice. Foods.

[38] Xu J., Wang X., Chen J., Chen S., Li Z., Liu H., Bai Y., Zhi F. (2020). Embryonic stem cell-derived mesenchymal stem cells promote colon epithelial integrity and regeneration by elevating circulating IGF-1 in colitis mice. Theranostics.

[39] Wang Y., Zhang B., Liu S., Xu E., Wang Z. (2023). The traditional herb Sargentodoxa cuneata alleviates DSS-induced colitis by attenuating epithelial barrier damage via blocking necroptotic signaling. J. Ethnopharmacol..

[40] Wells J.M., Brummer R.J., Derrien M., MacDonald T.T., Troost F., Cani P.D., Theodorou V., Dekker J., Meheust A., de Vos W.M. (2017). Homeostasis of the gut barrier and potential biomarkers. Am. J. Physiol. Gastrointest. Liver Physiol..

[41] Wang K., Wu L.Y., Dou C.Z., Guan X., Wu H.G., Liu H.R. (2016). Research Advance in Intestinal Mucosal Barrier and Pathogenesis of Crohn’s Disease. Gastroenterol. Res. Pract..

[42] Seo K., Seo J., Yeun J., Choi H., Kim Y.I., Chang S.Y. (2021). The role of mucosal barriers in human gut health. Arch. Pharm. Res..

[43] Dokladny K., Zuhl M.N., Moseley P.L. (2016). Intestinal epithelial barrier function and tight junction proteins with heat and exercise. J. Appl. Physiol..

[44] Feng Y., Chen S., Song Y., Liu S., Duan Y., Cai M., Kong T., Zhang H. (2023). A novel *Sagittaria sagittifolia* L. polysaccharides mitigate DSS-induced colitis via modulation of gut microbiota and MAPK/NF-kappaB signaling pathways. Int. J. Biol. Macromol..

[45] Wang P., Cai M., Yang K., Sun P., Xu J., Li Z., Tian B. (2023). Phenolics from Dendrobium officinale Leaf Ameliorate Dextran Sulfate Sodium-Induced Chronic Colitis by Regulating Gut Microbiota and Intestinal Barrier. J. Agric. Food Chem..

[46] Zhou Y., Xu Z.Z., He Y., Yang Y., Liu L., Lin Q., Nie Y., Li M., Zhi F., Liu S. (2018). Gut Microbiota Offers Universal Biomarkers across Ethnicity in Inflammatory Bowel Disease Diagnosis and Infliximab Response Prediction. mSystems.

[47] Huan Q., Peng J., Chang Y., Zhang Q., Xing T., Jiang D., Chen W., Shen X., Bian Z., Xiao H. (2023). Activation of P2Y1R impedes intestinal mucosa repair during colitis. Int. J. Biol. Sci..

[48] Wen Y., Tan L., Chen S., Wu N., Yao Y., Xu L., Xu M., Zhao Y., Tu Y. (2023). Egg yolk phosphatidylcholine alleviates DSS-induced colitis in BALB/c mice. Food Funct..

[49] Chen Y., Yang B., Stanton C., Ross R.P., Zhao J., Zhang H., Chen W. (2021). Bifidobacterium pseudocatenulatum Ameliorates DSS-Induced Colitis by Maintaining Intestinal Mechanical Barrier, Blocking Proinflammatory Cytokines, Inhibiting TLR4/NF-kappaB Signaling, and Altering Gut Microbiota. J. Agric. Food Chem..

[50] Li B., Du P., Du Y., Zhao D., Cai Y., Yang Q., Guo Z. (2021). Luteolin alleviates inflammation and modulates gut microbiota in ulcerative colitis rats. Life Sci..

[51] Wu Y., Ran L., Yang Y., Gao X., Peng M., Liu S., Sun L., Wan J., Wang Y., Yang K. (2023). Deferasirox alleviates DSS-induced ulcerative colitis in mice by inhibiting ferroptosis and improving intestinal microbiota. Life Sci..

[52] Li X., Wu X., Wang Q., Xu W., Zhao Q., Xu N., Hu X., Ye Z., Yu S., Liu J. (2022). Sanguinarine ameliorates DSS induced ulcerative colitis by inhibiting NLRP3 inflammasome activation and modulating intestinal microbiota in C57BL/6 mice. Phytomedicine.

[53] Eksteen B., Walker L.S., Adams D.H. (2005). Immune regulation and colitis: Suppression of acute inflammation allows the development of chronic inflammatory bowel disease. Gut.

[54] Tang Y., Shi Y., Gao Y., Xu X., Han T., Li J., Liu C. (2019). Oxytocin system alleviates intestinal inflammation by regulating macrophages polarization in experimental colitis. Clin. Sci..

[55] Lissner D., Schumann M., Batra A., Kredel L.I., Kuhl A.A., Erben U., May C., Schulzke J.D., Siegmund B. (2015). Monocyte and M1 Macrophage-induced Barrier Defect Contributes to Chronic Intestinal Inflammation in IBD. Inflamm. Bowel Dis..

[56] Liu Y., Chen K., Wang C., Gong W., Yoshimura T., Liu M., Wang J.M. (2013). Cell surface receptor FPR2 promotes antitumor host defense by limiting M2 polarization of macrophages. Cancer Res..

[57] Trojan E., Tylek K., Leskiewicz M., Lason W., Brandenburg L.O., Leopoldo M., Lacivita E., Basta-Kaim A. (2021). The N-Formyl Peptide Receptor 2 (FPR2) Agonist MR-39 Exhibits Anti-Inflammatory Activity in LPS-Stimulated Organotypic Hippocampal Cultures. Cells.

